# Retrospective Analysis of the Clinical Characteristics of *Candida auris* Infection Worldwide From 2009 to 2020

**DOI:** 10.3389/fmicb.2021.658329

**Published:** 2021-05-20

**Authors:** Shan Hu, Feilong Zhu, Weiwei Jiang, Yuehua Wang, Yongqiang Quan, Guoming Zhang, Feng Gu, Ying Yang

**Affiliations:** ^1^Department of Biotechnology, Beijing Institute of Radiation Medicine, Beijing Key Laboratory of New Molecular Diagnosis Technologies for Infectious Diseases, Beijing, China; ^2^Department of Laboratory Medicine, Xuzhou Tumor Hospital, Xuzhou, China; ^3^The Affiliated Xuzhou Rehabilitation Hospital of Xuzhou Medical University, Xuzhou Rehabilitation Hospital, Xuzhou, China; ^4^Shanghai Key Laboratory of Molecular Medical Mycology, Shanghai Institute of Mycology, Shanghai Changzheng Hospital, Second Military Medical University, Shanghai, China; ^5^Department of Life Science, Hebei University, Baoding, China; ^6^Department of Nephrology, Affiliated Huaihai Hospital of Xuzhou Medical University, Xuzhou, China; ^7^Department of Laboratory Medicine, Shuyang People’s Hospital, The Affiliated Shuyang Hospital of Xuzhou Medical University, Shuyang, China

**Keywords:** *Candida auris*, infection characteristics, underlying disease, drug resistance, risk factor for mortality

## Abstract

**Introduction:**

*Candida auris* is an emerging multidrug-resistant fungus that may cause infections with a high mortality rate. The first case of *C. auris* infection was reported in 2009 and infections have been reported in 44 countries. The fungus now represents a major global public health threat. We analyzed cases from the emergence of *C. auris* infections up until the end of 2020. It is hoped that the results of this analysis will raise awareness in scientists to promote protection and control research pertaining to this pathogen.

**Methods:**

PubMed and Web of Science databases were searched for all papers related to *C. auris* infections up until December 31, 2020. We sorted and organized these data into the following categories: date of publication, patient age and sex, underlying diseases, risk factors for infection, patient mortality information, drug sensitivity information of *C. auris* isolates, and genetic classification. The χ^2^ test was used to screen for factors that may affect patient mortality.

**Results:**

A total of 912 patients were included in the analysis. There’s a higher proportion of men and a high proportion of patients were premature babies and elderly people. The proportions of patients with underlying diseases such as diabetes, kidney disease, trauma, and ear disease were also high. More than half of patients had a history of central venous catheter use and a history of broad-spectrum antibiotic use. The χ^2^ test revealed that only kidney disease (*P* < 0.05) was an important risk factor for mortality in *C. auris-*infected patients.

**Conclusions:**

A comprehensive understanding of *C. auris* was achieved following this retrospective analysis, including the characteristics of *C. auris*-infected patients. In recent years, increasing numbers of multidrug-resistant *C. auris* isolates have been identified, and the high mortality rates associated with infection merit greater attention from the medical world.

## Introduction

Since 2009, when the first *Candida auris* infection case was reported in Japan (Satoh et al., 2009), until December 31, 2020, *C. auris* infections have been reported in 44 countries globally (Centers for Disease Control and Prevention, 2021). In April 2019, the *New York Times* reported a multidrug-resistant fungal infection outbreak in many parts of the United States. The causative agent of this outbreak was *C. auris*; however, the aforementioned article referred to the infections as “mysterious infections” and deemed the outbreak an “urgent threat.” Nearly 50% of infected individuals died within 90 days and the outbreak subsequently attracted widespread global attention (The New York Times, 2019). Because of the associated high mortality rates, *C. auris* can now be considered a major global public health threat (Meis and Chowdhary, 2018).

Before 2009, *C. auris* was thought to be a rarely observed microorganism. It is now deemed an emerging human pathogen (Rhodes and Fisher, 2019). *C. auris* can colonize different sites, such as the skin, axilla, nose, and groin, and is transmitted by contact or through feces. In addition, the pathogen can survive on inanimate object surfaces for more than 7 days (Calvo et al., 2016; Biswal et al., 2017). Therefore, hospital beds, sphygmometers, thermometers, and other reusable equipment are potential infection sources for inpatients. This has resulted in relatively high rates of *C. auris* transmission in inpatients, particularly in ICU rooms (Madder et al., 2017; Ku et al., 2018). This latter phenomenon has led to a vicious cycle of acquisition, transmission, and infection (Cortegiani et al., 2019). It is difficult to eradicate *C. auris* once it has colonized a patient, and colonization of patients can last for three or more continuous months (Jeffery-Smith et al., 2018). Even though many countries have implemented infection prevention and control (IPC) strategies, *C. auris* transmission is still a problem that warrants attention.

The appearance of multidrug resistance (MDR) in *C. auris* is another problem that has attracted global attention in recent years (Kathuria et al., 2015). The sensitivity of many *C. auris* isolates toward fluconazole has decreased and associated strains have developed varying degrees of resistance to other antifungal drugs (Chowdhary et al., 2017). Public health guidelines recommend that echinocandins (micafungin, caspofungin, and anidulafungin) should be used as first-line treatments for *C. auris* infections (Chowdhary et al., 2016; Kim Hutchings Uke, 2018). However, the continuous evolution of *C. auris* has resulted in echinocandin resistance. Indeed, the minimum inhibitory concentrations (MIC) of azoles, amphotericin B, and echinocandin toward some isolates have even increased (Rhodes et al., 2018). Therefore, close attention to the evolution of *C. auris* is required.

Traditional identification techniques cannot be used to identify *C. auris*. Therefore, the prevalence of *C. auris* infections in the global population remains unknown. *C. auris* has resulted in an “invisible pandemic” due to its broad nosocomial infection range (Jeffery-Smith et al., 2018). Thus, it is extremely important to comprehensively understand the characteristics of *C. auris* itself along with the patients that the fungus infects. With this in mind, we collected papers since the first report of *C. auris* and extracted information pertaining to the patients’ age and sex, underlying diseases, possible risk factors that may induce infection, genetic phenotype classification of *C. auris* isolates, drug susceptibility information, and drug resistance loci. We compiled this information and conducted a statistical analysis to identify new infection-related characteristics. The mortality rate of *C. auris* infection in patients is an important factor that threatens global public health (Meis and Chowdhary, 2018). Therefore, this study employed a conservative regression model to determine the effects of several factors on patient mortality. We believe that this analysis and summary of the relevant data will result in a greater public understanding of *C. auris* infections, thereby enabling scientists to focus greater attention on the evolution of multidrug resistance in *C. auris*.

## Materials and Methods

### Case Inclusion

PubMed and Web of Science were used to conduct the literature search using the following search criteria: 1. titles or abstracts containing the keyword *C. auris*; and 2. time range with an unlimited start date and an end date of December 31, 2020. A total of 580 papers were identified through the literature search. Experimental study papers were excluded while case reports were selected for the analysis. Of these, 146 papers reporting *C. auris* cases were included; these reports involved nearly 6,000 patients.

The papers were organized and analyzed after case screening. First, repeated cases were excluded: suspected repetitive cases from the same year and country were excluded based on isolate number, detection time, and detection institution; cases with incomplete information were excluded; and cases identified as infection caused by colonized *C. auris* were excluded. The data of 912 cases were included in the statistical analysis.

### Data Entry

The following information required for analysis was input:

In order to ensure the accuracy of analysis, each dataset required review by at least three members of the team before it was accepted. In this study, the Beijing Key Laboratory for Molecular Diagnostics of Infectious Diseases was deemed the leading site and it established a research team consisting of one clinician, two laboratory physicians, one medical mycology researcher, and one pharmacology researcher.

### Data Organization and Statistical Analysis

In this study, underlying diseases predominantly included systemic diseases, comorbid infections, and organ diseases; these were further divided into 52 subcategories. Suspected patients were not included in the statistics.

In order to avoid the repetition of statistical analyses pertaining to drug sensitivity data, the most recent data for an isolate were used when the same method was employed to analyze more than one drug sensitivity experiment. Interestingly, the MIC values for the most recent results generated from drug sensitivity experiments in all papers were the highest. When the broth method and standard commercial reagents were both used for drug sensitivity experiments for the same isolate, the drug sensitivity results obtained using standard commercial reagents were chosen for analysis. When different standard commercial drug sensitivity test systems (VITEK^®^2 and Sensititre^®^ YeastOne^®^ were used in this study) were used for the same isolate, the test results from the VITEK^®^2 system were selected as the inter-laboratory consistency of VITEK^®^2 was higher than that for Sensititre^®^ YeastOne^®^ (Farina et al., 2011).

The mortality rate of *C. auris* infection is an important factor that threatens global public health. Hence, in this study we felt that it was important to screen for factors that affected patient mortality. We first extracted the information from all the cases in the literature, excluded the cases that missed important data, and included the cases with complete data in the analysis. The χ^2^ test was used for univariate analysis of the final complete cases to identify indicators that had a significant association with the outcome (Peduzzi et al., 1996). If the *P*-values of multiple indicators were less than 0.05, these indicators were included in a multivariate regression model, and P-value correction was carried out to find the significant indicators under the influence of multiple factors (Hosmer et al., 2013). Otherwise, multivariate analysis was not performed (Mejia-Chew et al., 2019).

## Results

### Initial Infection Reports in Different Countries

**FIGURE 1:**
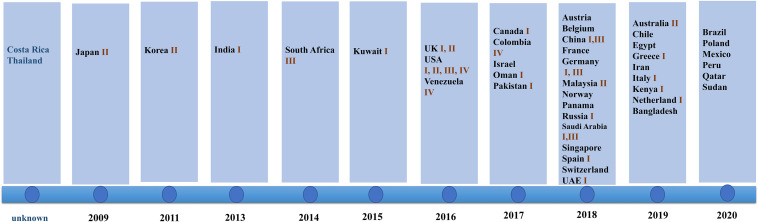
Date of first reports and *Candida* genetic classification based on the country of origin including South Asia (clade I), East Asia (clade II), South Africa (clade III), and South America (clade IV). In 2018, the highest number of countries reported infection for the first time. Clade I was reported in 17 countries, clade III was reported in eight countries, clade II was reported in five countries, and clade IV was reported in three countries.

Since 2009 when *C. auris* was first isolated from an auditory canal of a patient in Japan, infections have also been reported in South Korea (Oh et al., 2011), India (Chowdhary et al., 2013), South Africa (Rindidzani et al., 2014), and Kuwait (Emara et al., 2015). After 2016, the number of countries reporting their first cases of *C. auris* infection began to increase. In 2018, 14 countries reported *C. auris* infections for the first time. This was the highest number of countries reporting infections for the first time in 12 years (shown in Figure 1).

### Genetic Classification of Isolates Detected in Various Countries

The isolates detected by various countries were classified by whole genome sequencing data into South Asia strains (clade I), East Asia strains (clade II), South Africa strains (clade III), and South America strains (clade IV). The South Asia strains (clade I) were the most prevalent (17 countries), followed by the South Africa strains (clade III), which were observed in eight countries. Only five and three countries reported the presence of the East Asia strains (clade II) and the South America strains (IV), respectively. Both South Asia and South Africa strains simultaneously occurred in China, Germany, United Kingdom, and Saudi Arabia. All four clades appeared in the United States, a phenomenon that may be due to the large population movements. In addition, an isolate identified in Iran in 2019 may represent a potential clade V strain (shown in Figure 1; Nancy et al., 2019).

### Patient Sex and Age Distribution

**FIGURE 2:**
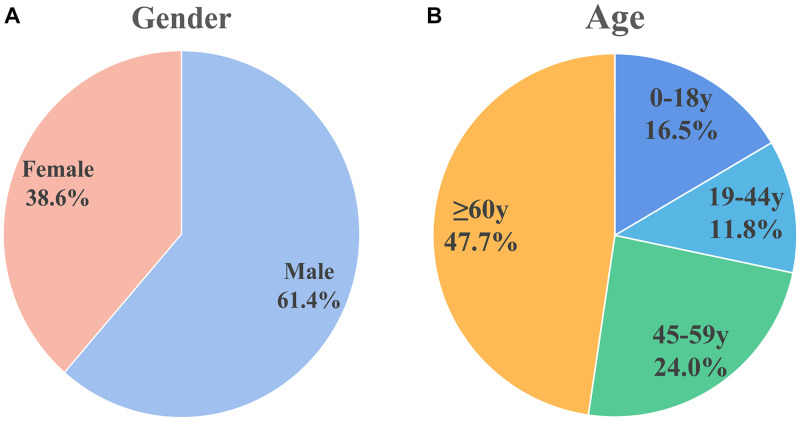
Sex and age distribution of patients included in the analysis. **(A)** Sex distribution: males (61.4%) and females (38.6%). **(B)** Age distribution: 0–18 years (16.5%), 19–44 years (11.8%), 45–59 years (24.0%), and ≥60 years (47.7%). Male patients and older patients are more susceptible.

For the included patients, sex was mentioned for 827 patients, of which 508 (61.4%) were male and 319 (38.6%) were female, there’s a higher proportion of men. This is shown in Figure 2A.

The World Health Organization age group classification criteria (Hosmer et al., 2013) were used to divide patients into children (0–18 years), adolescents (19–44 years), middle-aged (45–59 years), and elderly (≥ 60 years). Figure 2B shows the age distribution of 279 cases in which age was mentioned. Approximately half of the population (*n* = 133, 47.7%) were elderly. Thirty-two of the patients were infants less than 1 month old; 26 of the 32 (81.25% of the infant population) patients were premature infants.

### Underlying Disease and Infected Population

Underlying diseases were mentioned for 748 patients, of which the highest proportion of underlying diseases were diabetes, kidney disease (some had diabetic nephropathy), trauma, and ear disease. It should be noted that 10 patients also had COVID-19 (as shown in Table 1).

**TABLE 1:** Underlying diseases in *C. auris-*infected patients.

Underlying disease	Number of patients	Proportion of patients
Diabetes	149	19.9%
Kidney disease	138	18.4%
Trauma	91	12.2%
Ear disease	74	9.9%
Lung disease	63	8.4%
Hypertension	58	7.8%
Tumor	53	7.1%
Brain disease	47	6.3%
Brain disease	46	6.1%
Liver disease	25	3.3%
Intestinal disease	23	3.1%
Urinary tract diseases	21	2.8%
Hematologic disease	15	2.0%
Nervous system disease	13	1.7%
Acquired immunodeficiency syndrome (AIDS)	12	1.6%
Vasculopathy	10	1.3%
COVID-19	10	1.3%
Chemotherapy	9	1.2%
Thyroid disorder	9	1.2%
Pancreatic disease	9	1.2%
Other diseases	71	9.5%

*Other diseases are underlying disease categories with proportion of patients < 1%.*

### Risk Factors That May Lead to Infection and Infected Population

Risk factors for infection were mentioned for 773 patients (Table 2). Greater than 50% of patients had a history of broad-spectrum antibiotic use and a history of central venous catheter use. These factors represent high risk factors for *C. auris* infection and are consistent with the risk factors espoused by global experts in relation to *C. auris* emergence and transmission (Hosmer et al., 2013).

**TABLE 2:** Risk factors involved with *C. auris-*infected patients.

Risk factor	Number of patients	Proportion of patients
Broad-spectrum antibiotic treatment	432	55.9%
Central venous catheter	426	55.1%
ICU	378	48.9%
Urinary catheter	294	38.0%
Surgery	287	37.1%
Treatment with antifungal drugs before testing positive	216	27.9%
Mechanical ventilation	204	26.4%
Immunosuppressant	134	17.3%
Parenteral nutrition	108	14.0%
Steroid treatment	81	10.5%
Blood transfusion	45	5.8%
Neutropenia	13	1.7%

### Drug-Resistant *Candida auris* Strains

**FIGURE 3:**
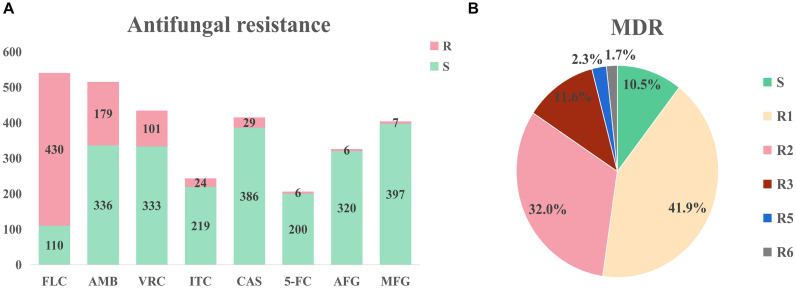
Drug resistance of *Candida auris* isolates. **(A)** Drug resistance of *Candida auris* against eight common antifungals. The isolates had severe resistance to azoles and amphotericin B, and low resistance to echinomycin. **(B)** Drug resistance of *Candida auris* isolates. MDR: multi-drug resistance. S: sensitive, R1-R6: drug resistance status. Isolates had severe multidrug resistance, with some isolates exhibiting resistance to six antifungal agents.

Large differences in efficacy were observed for antifungal drugs in relation to the treatment of infection of different *C. auris* isolates (Chowdhary et al., 2017). The United States Centers for Disease Control (CDC) defined conservative cutoff points to determine whether *C. auris* is resistant to antifungals: fluconazole (FLC): 32 μg/mL, amphotericin B (AMB): 2 μg/mL, micafungin (MFG): 4 μg/mL, and caspofungin (CAS): 2 μg/mL.^2^ In another study by the United States CDC, break points for voriconazole (VRC, 2 μg/mL), anidulafungin (AFG, 4 μg/mL), and flucytosine (5-FC, 32 μg/mL) were established. A MIC < cutoff was defined as sensitive while a MIC ≥ cutoff was defined as resistant (Lockhart et al., 2016). Among the isolates that were included in the analysis, drug resistance information was available for 544 isolates; the proportion of isolates exhibiting FLC resistance was the highest (79.6%), while isolates exhibiting AMB (34.8%%) and VRC (23.3%) resistance were the second and third most prevalent, respectively (Figure 3A). Among the VRC-resistant isolates, 18 Venezuelan isolates reported by Calvo et al. (2016). and eight United States isolates reported by Park et al. (2019) were completely resistant to VRC. Among the AMB-resistant isolates, five Qatar isolates that were reported by A Shaukat et al. (2020) were completely resistant to AMB.

Minimum inhibitory concentrations information was accessible for 172 isolates in the literature. This information was used to determine drug resistance (Figure 3B). Among these isolates, 18 isolates (10.5%) were sensitive, 72 isolates (41.9%) were resistant to one antifungal, 55 isolates (32.0%) were resistant to two antifungals, 20 isolates (11.6%) were resistant to three antifungals, four isolates (2.3%) were resistant to five antifungals, and three isolates (1.7%) were resistant to six antifungals. It should be noted that sensitive isolates were retrieved from six dead patients and the only one sensitive isolate was isolated from a surviving patient. Three isolates were resistant to six drugs (FLC, VRC, AMB, CAS, MFG, and AFG) and these isolates were obtained from three dead patients from the United States (Ostrowsky et al., 2020). Currently, echinocandin is the first-line drug for *C. auris* infection (Chowdhary et al., 2016; Kim Hutchings Uke, 2018).

### Drug Resistance Loci

Among the isolates reported to date, the major drug resistance loci for azoles are ERG11, CDR1, and MDR1 (Healey et al., 2018; Kordalewska et al., 2018; Rybak et al., 2019); the major drug resistance loci for AMB are ERG 2, 3, 5, 6, or 11 (Rhodes et al., 2018); and the major drug resistance locus for echinocandin is FKS11 (Chowdhary et al., 2018; Hou et al., 2019). As part of a mechanistic analysis of drug resistance toward 5FC, Rhodes et al. (2018) sequenced the entire genome of 5FC-resistant *C. auris* and found an amino acid substitution in F211I in the FUR1 gene. However, further studies are required.

### Mortality Rate

A total of 476 cases reported whether patients died or survived upon discharge. Among the latter cases, 226 died and the mortality rate was 47.5%. Major causes of death that were mentioned included sepsis, septic shock, and multiorgan failure.

### Risk Factor for Mortality

A total of 167 related cases were collected by literature retrieval, and 144 cases were included for analysis after excluding those missing important information. Based on the above univariate analysis, it was found that kidney disease was an important factor associated with mortality, with *P* = 0.029 for patients with kidney disease (Table 3). The other indexes had no significant associations with mortality. Therefore, multivariate logistic regression model was not performed (Palazón-Bru et al., 2017).

**TABLE 3:** χ^2^ test screening of single factors that may be associated with patient mortality.

Factor		Number of deaths	Number of survived patients	*P*-value
Sex	Male	46	47	0.340
	Female	21	30	
Sepsis	Absent	55	69	0.193
	Present	12	8	
Fever	Absent	59	67	0.850
	Present	8	10	
Ear disease	Absent	67	76	0.349
	Present	0	1	
Diabetes	Absent	46	54	0.848
	Present	21	23	
Hypertension	Absent	55	62	0.810
	Present	12	15	
Hypotension	Absent	66	76	0.921
	Present	1	1	
Hyperlipidemia	Absent	66	76	0.921
	Present	1	1	
Lung disease	Absent	46	61	0.148
	Present	21	12	
Tracheal disease	Absent	65	76	0.480
	Present	2	1	
Intestinal disease	Absent	60	70	0.784
	Present	7	7	
Kidney disease	Absent	41	60	0.029
	Present	26	17	
Liver disease	Absent	59	73	0.144
	Present	8	4	
Gallbladder disease	Absent	66	76	0.921
	Present	1	1	
Tumor	Absent	56	68	0.413
	Present	11	9	
Chemotherapy	Absent	65	76	0.480
	Present	2	1	
Nasopharyngeal disease	Absent	65	77	0.127
	Present	2	0	
Laryngeal disease	Absent	66	77	0.282
	Present	1	0	
Hematologic disease	Absent	63	73	0.839
	Present	4	4	
Heart disease	Absent	52	66	0.207
	Present	15	11	
Neuropathy	Absent	73	87	0.127
	Present	65	77	
	Present	2	0	
Vasculopathy	Absent	64	73	0.842
	Present	3	4	
Abnormal coagulation function	Absent	65	76	0.480
	Present	2	1	
Thyroid disorder	Absent	63	76	0.127
	Present	4	1	
Drug abuse	Absent	67	76	0.349
	Present	0	1	
AIDS	Absent	65	75	0.888
	Present	2	2	
	Present	0	0	
	Present	1	0	
COVID-19	Absent	61	73	0.376
	Present	6	4	
Pancreatic Disease	Absent	67	75	0.184
	Present	0	2	
Bone marrow disease	Absent	65	75	0.888
	Present	2	2	
Oral disease	Absent	67	76	0.349
	Present	0	1	
Urinary tract disease	Absent	64	74	0.862
	Present	3	3	
Endometrial disease	Absent	67	76	0.349
	Present	0	1	
Cytomegalovirus (CMV)	Absent	73	86	0.480
	Present	65	76	
	Present	2	1	
Psychiatric disorder	Absent	66	75	0.643
	Present	1	2	
Psoriasis	Absent	66	77	0.282
	Present	1	0	
Pressure ulcer	Absent	64	74	0.842
	Present	3	3	
Asthma	Absent	66	77	0.282
	Present	1	0	
Adrenal glands	Absent	67	76	0.349
	Present	0	1	
Prostate gland	Absent	66	77	0.282
	Present	1	0	
Breasts	Absent	75	86	0.349
	Present	67	76	
	Present	0	1	
Bone fracture	Absent	74	66	0.134
	Present	66	72	
	Present	1	5	
Trauma	Absent	62	73	0.575
	Present	5	4	
Age #		64(31–74)	53(32.5–72)	0.172

*#Non-normal distribution after normality test was carried out and expressed as median (interquartile range).*

## Discussion

*Candida auris* is an emerging pathogen and there is currently only a limited understanding of the mechanisms and risk factors that underpin associated infections. Hence, further studies are required to help scientists better understand this pathogen. During the early stages of emergence of a pathogen, information release along with medical and public health awareness are extremely important.

In this analysis of collected literature, incomplete information was available for many patients. The main reason for these gaps in information relate to the fact that *C. auris* isolates from these patients were not isolated during hospitalization but a few years later by public health institutions and research institutions during retrospective screening of samples. Many medical institutions still rely on culture to identify microorganisms (Navalkele et al., 2017). *C. auris* is closely related to *Candida haemulonii* and *Candida pseudohaemulonii*. They are extremely close in phylogeny, and it is difficult to distinguish them by phenotype. Thus, traditional identification methods such as Vitek 2 and API 20CAUX tend to incorrectly identify *C. auris* as these two close relatives (Kathuria et al., 2015). Although the development of matrix-assisted laser desorption/ionization time-of-flight mass spectrometry (MALDI-TOF MS) (Vatanshenassan et al., 2019) and sequencing (Muñoz et al., 2018) strategies have helped in the rapid and accurate diagnosis of *C. auris*, most parts of the world do not have the infrastructure to carry out these techniques. In addition, it is likely that there is a large volume of unpublished data pertaining to *C. auris* infections and the number of infected patients may be far higher than that reported in the literature. Therefore, this is an “invisible pandemic” (Jeffery-Smith et al., 2018).

In this retrospective analysis, we found that there’s a higher proportion of male patients. The proportion of *C. auris* patients with diabetes, kidney disease, trauma, and ear disease is high. Jung et al. (Mejia-Chew et al., 2019) reported that 69 out of 79 *C. auris* infection patients had ear disease and their symptoms were relatively mild. Therefore, otolaryngology outpatient departments should be highly vigilant for *C. auris* infections. Among the *C. auris* patient population, 18.4% had kidney disease. Most of these patients had chronic kidney disease or nephrotic syndrome. More importantly, kidney disease is also an important risk factor that affects mortality in *C. auris* infection patients. Nephrologists should be extremely vigilant for *C. auris* infection. The reasons for the association of kidney disease with mortality may include the frequent use of immunosuppressants in the treatment process of patients with kidney disease; (Wang et al., 2019) the fact that most patients with kidney disease have low protein and malnutrition, which leads to a decline in immunity (Rhee et al., 2018); the fact that patients with kidney disease often suffer from diabetes (Alicic et al., 2017); and the dialysis treatments required by many patients with kidney disease (Collister et al., 2017). As the high infection rate in kidney disease patients is specific to *C. auris* compared with other *Candida* species, nephrologists in many regions do not have a high awareness of *C. auris* infections. Many nephrologists do not completely understand *C. auris*. Therefore, it is important that *C. auris-*related information be disseminated to various medical units, particularly nephrology departments, which can be extremely important in the early diagnosis of *C. auris* infections. In addition, although ICUs have been a focus of attention since *C. auris* was first reported, overall vigilance in ICUs is still not very strong and there is a need to strengthen knowledge gaps pertaining to prevention, diagnosis, and treatment knowledge.

Fluconazole exhibits broad-spectrum antifungal activity, good efficacy, a low incidence of adverse reactions, and appropriate plasma concentrations for long durations (Agrawal et al., 1996). *C. auris* has a high FLC resistance rate and the overall drug resistance ratio has been as high as 79.6% over the last 12 years. Thus, FLC is not recommended as an empiric drug therapy when treatment is urgent and drug sensitivity results have yet to be released for patients who either have been infected with *C. auris* or are suspected to have been infected with *C. auris*.

We did identify some areas for attention after an in-depth analysis of multidrug resistance in *C. auris*. Among the isolates for which patient mortality could be determined, sensitive ones were isolated from six dead patients and only one sensitive isolate was obtained from a surviving patient. We analyzed the possible reasons for the dearth of information pertaining to isolates causing mortality and speculated that virulence and mortality rates are higher when the *C. auris* isolate is sensitive (Tan et al., 2019; Lara et al., 2020). Sensitive isolates can be treated with antifungal drugs, thus clinical outcomes can be changed if the patients are diagnosed early and treated in a timely manner.

In addition, among the acquired data, three isolates that were resistant to six drugs were obtained and isolated from patients from the United States, where the outcome was death. Therefore, when multidrug resistance in *C. auris* gradually evolves, there will be situations where patients cannot be treated by drugs and death will occur. Echinocandin is currently the recommended treatment for *C. auris* (Kordalewska et al., 2018). If *C. auris* gradually evolves, more and more strains will develop echinocandin resistance and this may lead to an increase in the mortality rate of patients with *C. auris* infections. Although SCY-078 (Larkin et al., 2017) and other new drugs have shown some promise, they have not been widely used in clinical practice. It is especially important to develop new drugs to combat high levels of echinocandin resistance.

There are some limitations pertaining to this study. As this is a retrospective analysis of published studies, there may be some original studies where the authors believed the associated conditions were not important and some of the risk factors were not mentioned. Thus, these papers were not included in this current analysis. As there was only a limited number of patients with certain diseases, there might be a selection bias in this study.

## Conclusion

This retrospective analysis provides us with a greater understanding of *C. auris*, along with some of the main characteristics associated with *C. auris*-infected patients. In addition, an ever-increasing number of multidrug resistant isolates have been identified and we should be vigilant in relation to the evolution of drug resistance in *C. auris.* It is important that we comprehensively improve diagnosis and treatment awareness for *C. auris*. We should actively develop early diagnostic techniques along with safe and effective drugs for the treatment of *C. auris* infections.

## Data Availability Statement

The original contributions presented in the study are included in the article/supplementary material, further inquiries can be directed to the corresponding author/s.

## Author Contributions

YY and FG conceived and designed the study. SH, WJ, YW, and YQ collated and collected data. FZ and GZ did the statistical analysis. SH, FZ, WJ, and YY drafted the manuscript. All authors played a significant role in data collection and analysis.

## Conflict of Interest

The authors declare that the research was conducted in the absence of any commercial or financial relationships that could be construed as a potential conflict of interest.
